# Mcm2 phosphorylation and the response to replicative stress

**DOI:** 10.1186/1471-2156-13-36

**Published:** 2012-05-07

**Authors:** Brent E Stead, Christopher J Brandl, Matthew K Sandre, Megan J Davey

**Affiliations:** 1 Department of Biochemistry, Schulich School of Medicine & Dentistry, University of Western Ontario, London, ON, N6A 5C1, Canada

## Abstract

**Background:**

The replicative helicase in eukaryotic cells is comprised of minichromosome maintenance (Mcm) proteins 2 through 7 (Mcm2-7) and is a key target for regulation of cell proliferation. In addition, it is regulated in response to replicative stress. One of the protein kinases that targets Mcm2-7 is the Dbf4-dependent kinase Cdc7 (DDK). In a previous study, we showed that alanine mutations of the DDK phosphorylation sites at S164 and S170 in *Saccharomyces cerevisiae* Mcm2 result in sensitivity to caffeine and methyl methanesulfonate (MMS) leading us to suggest that DDK phosphorylation of Mcm2 is required in response to replicative stress.

**Results:**

We show here that a strain with the *mcm2* allele lacking DDK phosphorylation sites (*mcm2*_AA_) is also sensitive to the ribonucleotide reductase inhibitor, hydroxyurea (HU) and to the base analogue 5-fluorouracil (5-FU) but not the radiomimetic drug, phleomycin. We screened the budding yeast non-essential deletion collection for synthetic lethal interactions with *mcm2*_AA_ and isolated deletions that include genes involved in the control of genome integrity and oxidative stress. In addition, the spontaneous mutation rate, as measured by mutations in *CAN1*, was increased in the *mcm2*_AA_ strain compared to wild type, whereas with a phosphomimetic allele (*mcm2*_EE_) the mutation rate was decreased. These results led to the idea that the *mcm2*_AA_ strain is unable to respond properly to DNA damage. We examined this by screening the deletion collection for suppressors of the caffeine sensitivity of *mcm2*_AA_. Deletions that decrease spontaneous DNA damage, increase homologous recombination or slow replication forks were isolated. Many of the suppressors of caffeine sensitivity suppressed other phenotypes of *mcm2*_AA_ including sensitivity to genotoxic drugs, the increased frequency of cells with RPA foci and the increased mutation rate.

**Conclusions:**

Together these observations point to a role for DDK-mediated phosphorylation of Mcm2 in the response to replicative stress, including some forms of DNA damage. We suggest that phosphorylation of Mcm2 modulates Mcm2-7 activity resulting in the stabilization of replication forks in response to replicative stress.

## Background

DNA replication is tightly regulated to ensure that genomes are copied once and only once per cell division cycle [1]. In addition, cells must respond to assaults that damage DNA and/or disrupt replication forks by preventing the initiation of DNA replication and stabilizing active replication forks [2]. One of the targets for these regulatory events is the replicative helicase that unwinds DNA at the replication fork [3-8]. The catalytic core of the replicative helicase in eukaryotic cells is a heterohexameric complex comprised of the minichromosome maintenance proteins 2 through 7 (Mcm2-7; [9,10]). Mcm2-7 activity is tightly controlled during the initiation of DNA replication and is targeted in response to replicative stress [3,6-8,11-14].

*In vivo*, Mcm2-7 functions within the CMG complex comprised of Cdc45, Mcm2-7 and the tetrameric GINS complex (Sld5, Psf1, Psf2 and Psf3) [11,12]. The catalytic activity for DNA unwinding resides in Mcm2-7 with Cdc45 and GINS playing roles in limiting Mcm2-7 activity to S phase and providing scaffolding functions within the replisome [11,15-17]. CMG is isolated from replicating yeast cells as part of the RPC (replisome progression complex) that includes the checkpoint protein Mrc1, the fork pausing complex Tof1-Csm3, the histone chaperone FACT and the sister chromatid cohesion factor, Ctf4 [16].

Mcm2-7 activity is also regulated by phosphorylation. A recent study from our laboratory showed that phosphorylation of *Saccharomyces cerevisiae* Mcm2 by the Dbf4-dependent kinase, Cdc7 (DDK) at S164 and S170 is important for a proper response to DNA damage [5]. Strains containing a non-phosphorylatable allele of *mcm2* (*mcm2*_AA_) grow similarly to wild type cells in normal growth conditions but are sensitive to the DNA alkylating agent, methyl methanesulfonate (MMS) and to caffeine. Caffeine is a purine analogue with pleiotropic effects. In general, caffeine inhibits PI3K-related kinases, which in yeast include TOR (Tor1 and Tor2), Mec1 and Tel1 [18-21]. TOR controls cell growth in response to nutrients and stress whereas Mec1 and Tel1 are both checkpoint kinases that also have roles in control of replication initiation (Mec1) and telomere maintenance (Tel1) [3,22-24].

Here, we show that in addition to MMS and caffeine, the *mcm2*_AA_ strain is sensitive to the ribonucleotide reductase inhibitor, hydroxyurea (HU) and the base analogue 5-fluorouracil (5-FU), but not phleomycin, a radiomimetic drug. The phosphomimetic glutamic acid substitutions at S164 and S170 suppress sensitivity to these drugs. We examined the genetic network within which *mcm2*_AA_ functions and found 9 deletions that have synthetic slow growth or lethal interactions with *mcm2*_AA_ and 16 deletions that suppress the caffeine sensitivity of *mcm2*_AA._ The identities of these gene deletions are consistent with a role for Mcm2 phosphorylation in the response to DNA damage and replicative stress and include two members of the RPC. A role in response to replicative stress is emphasized by the higher than wild type spontaneous mutation rate in the *mcm2*_AA_ strain and a lower than wild type mutation rate with the *mcm2*_EE_ phosphomimetic allele. Most of the gene deletions that suppressed the caffeine sensitivity of *mcm2*_AA_ also relieved other phenotypes of *mcm2*_AA_. We propose that phosphorylation of Mcm2 by DDK is required in response to replicative stress to stabilize Mcm2-7 at replication forks.

## Results

### Growth of *mcm2*_AA_ and *mcm2*_EE_ cells in the presence of replicative stress

**Figure 1:**
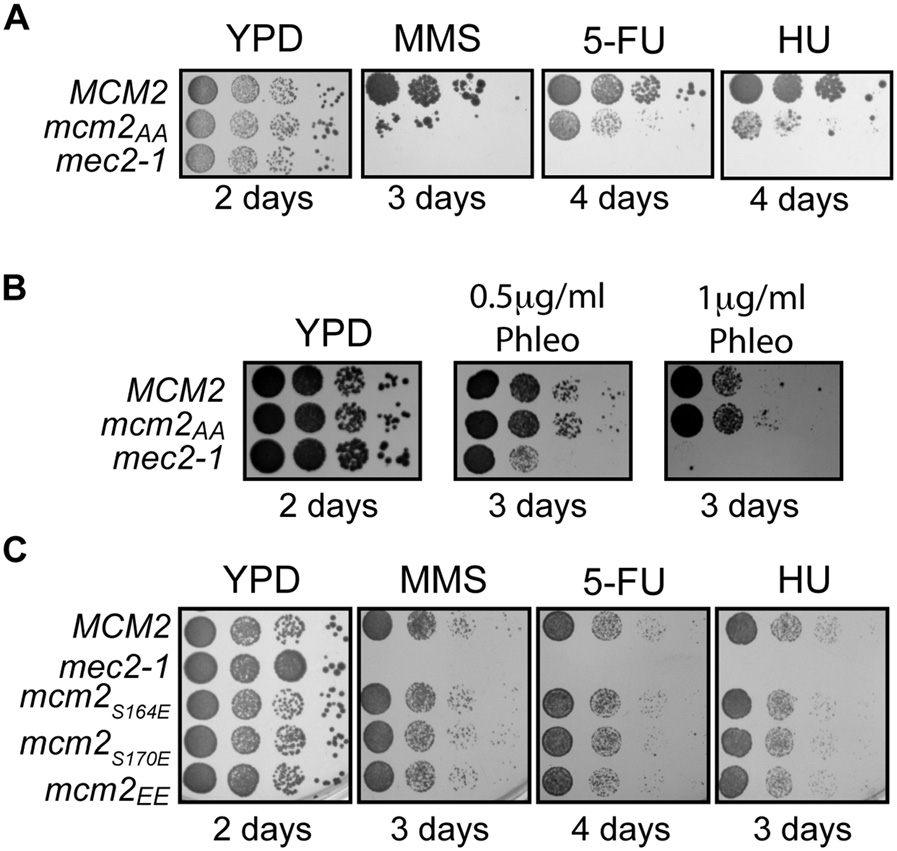
**Sensitivities of *mcm2 *alleles to genotoxic agents.** The growth of phosphorylation site mutations in *mcm2* was compared on plates containing genotoxic agents. The *mec2-1 *strain [24] was utilized to control for plate quality. **A)** The growth of *mcm2*_AA_ and *MCM2* strains was compared on YPD and YPD containing 0.03% MMS, 400 μM 5-FU or 200 mM HU by spotting 10-fold serial dilutions of the strains followed by growth at 30°C for the indicated times. **B)** Similarly, the growth of *mcm2*_AA_ and *MCM2* strains was compared on YPD with and without phleomycin. **C)** The growth of *mcm2*_EE_*mcm2*_S164E_*mcm2*_S170E_ and *MCM2* on YPD with 0.03% MMS, 400 μM 5-FU or 200 mM HU.

We examined the growth of the *mcm2*_AA_ strain on media containing agents that cause replicative stress (Figure 1A & B). The *mcm2*_AA_ strain had reduced growth relative to *MCM2* on YPD plates containing MMS or 5-FU (Figure 1A), but not on plates containing phleomycin (Figure 1B). These agents have different effects on DNA stability in budding yeast. MMS damages DNA by methylating guanines and adenines [25]. The effects of 5-fluorouracil in yeast are two-fold: it inhibits the pyrimidine biosynthesis pathway and results in misincorporation of uracil into nascent DNA [26]. Phleomycin is structurally similar to bleomycin, a radiomimetic drug that causes double stranded DNA breaks [27]. The *mcm2*_AA_ strain also grows poorly upon constant exposure to the ribonucleotide reductase inhibitor, hydroxyurea (HU), which interferes with the integrity of DNA replication forks and induces an S phase checkpoint [28,29].

We noted previously that Mcm2 in which S164 and S170 are altered to glutamic acids (*mcm2*_EE_) acts like a phosphomimic, allowing growth of cells in the presence of caffeine and MMS, and has the same activity *in vitro* as phospho-Mcm2 [5]. If phosphorylation of Mcm2 is required in response to 5-FU and HU, then *mcm2*_EE_ should be insensitive to these agents. As predicted, the *mcm2*_EE_ strain grew similarly to wild type cells in the presence of 5-FU and HU (Figure 1C). Substitution of Glu for S at position 164 or 170 also resulted in wild type growth consistent with the requirement to mutate both Ser to Ala to obtain a phenotype [5].

### Synthetic lethal/slow growth interactions with *mcm2*_AA_

The sensitivity of the *mcm2*_AA_ strain to caffeine, MMS, 5-FU and HU suggests that phosphorylation of Mcm2 is required in response to replicative stress. Furthermore, the increased frequency of RPA foci in these cells [5] suggests disruption of replication forks or an inability to respond to replicative stress [30-32]. If our model is correct, then mutations that increase genomic instability will be synthetic lethal or show slow growth with *mcm2*_AA_. After screening the *S. cerevisiae* non-essential deletion collection for synthetic lethal interactions with *mcm2*_AA_ and confirming the interactions by tetrad dissection, we found 8 gene deletions that result in no or slow growth when combined with *mcm2*_AA_ (Table 1 and Additional file 1). Three of the eight gene deletions that display synthetic lethal or slow growth interactions with *mcm2*_AA_ affect cell stress responses or cell cycle. In particular, the synthetic interaction of *mcm2*_AA_ with *chk1Δ*, a deletion in the gene encoding a checkpoint effector kinase is consistent with the idea that *mcm2*_AA_ is important in response to replicative stress. In addition, *ctf4Δsod1Δ* and *img1Δ* all lead to genomic instability or increase DNA damage [33-36] and their negative synthetic interactions with *mcm2*_AA_ support the idea that Mcm2 phosphorylation is important in response to DNA damage.

**Table 1:** **Synthetic lethal or slow growth interactions with *mcm2***_***AA***_

Gene	ORF	Function	GO	growth
*chk1Δ*	YBR274W	Checkpoint Kinase	1,2,5	lethal
*ctf4Δ*	YPR135W	Sister Chromatid Cohesion	1,2	lethal
*sod1Δ*	YJR104C	Response to oxygen radicals	1,6,8	slow
*bud23Δ*	YCR047C	Bud site selection	2,3,4	lethal
*pep3Δ*	YLR148W	Vesicular docking/Vacuolar biogenesis	3,8	lethal
*skn1Δ*	YGR143W	Sphingolipid biosynthesis	7,8	slow
*img1Δ*	YCR046C	Mitochondrial genome maintenance	9	lethal
*vma13Δ*	YPR036W	Subunit of Vacuolar ATPase	10	lethal

Genes are grouped by their GO terms as annotated in the *Saccharomyces cerevisiae* database [37]. Gene ontology: (1) Response to cell stress/chemical stimuli (2) cell cycle, (3) transport, (4) RNA metabolic process, (5) signalling process/protein modification process, (6) transcription, (7) carbohydrate metabolic process, (8) cell wall, membrane, & vesicle mediated transport, (9) mitochondrial organization, and (10) other.

### Rad53 is phosphorylated in the *mcm2*_AA_ strain

**Figure 2:**
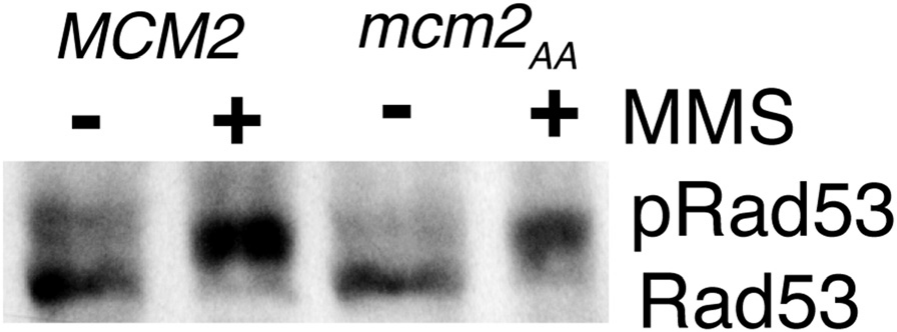
**Rad53 activation in the *mcm2 ***_AA _**strain.** Western blots of crude protein lysates of *mcm2*_AA_ or *MCM2* strains probed with anti-Rad53 antibody before and after treatment with MMS. Log phase cultures in YPD were grown for 2 hours at 30°C in the presence or absence of 0.02% MMS. After harvesting by centrifugation, protein was extracted with TCA, as described [38]. Approximately 20 μg of total protein was examined by Western blotting using anti-Rad53 antibody (Santa Cruz Biotechnology, SC-6749) as primary antibody followed by rabbit anti-goat IgG coupled to horseradish peroxidase (Sigma-Aldrich). The blots were visualised using the Supersignal West Pico chemiluminescence kit (Pierce) and x-ray film.

One potential role of Mcm2 phosphorylation in response to replicative stress is in the induction of a checkpoint signal leading to phosphorylation of Rad53, detected by decreased migration through SDS-PAGE. We examined Rad53 by Western blotting in the *MCM2* and *mcm2*_AA_ strains before and after treatment with 0.02% MMS, which triggers the S phase checkpoint. As seen in Figure 2, the migration of Rad53 is slower in the presence of MMS in both strains, suggesting that signalling in response to DNA damage is intact and that phosphorylation of Mcm2 is not required to activate checkpoint. We also tested for a genetic interaction between *mcm2*_AA_ and a checkpoint deficient allele of *RAD53* (*mec2-1*[24]). After mating the strains and generating spore progeny by tetrad dissection, none of the spore colonies contained both mutations indicating a synthetic lethal interaction, consistent with Mcm2 phosphorylation functioning in a parallel pathway to Rad53 ( Additional file 1).

### Increased mutation rate in *mcm2*_AA_ cells

If phosphorylation of Mcm2 is important in response to DNA damage and/or replicative stress, cells containing the non-phosphorylatable allele of *mcm2* (*mcm2*_AA_) would be predicted to accumulate mutations at a higher rate than cells with *MCM2* or *mcm2*_EE_. To test this, we utilized the *CAN1* forward mutation assay in which a mutation rate is determined from the number of canavanine resistant colonies that arise. *CAN1* encodes a transporter that enables the toxic compound canavanine to enter cells. If *CAN1* function is lost, then the cell is rendered resistant to canavanine. We grew *CAN1* strains containing different alleles of *mcm2* for several generations in liquid media without selection before determining the number of canavanine resistant colonies and the mutation rate using the method of the median [39,40]. The mutation rate was nearly two-fold higher in the *mcm2*_AA_ strain than with *MCM2* (5.5 x 10^-7^ v. 3.2 x 10^-7^), consistent with an inability to respond properly to spontaneous DNA damage in the absence of Mcm2 phosphorylation. Significantly, the mutation rate in the *mcm2*_EE_ strain was half that of the *MCM2* strain (1.6 x 10^-7^).

### Suppressors of the caffeine sensitivity of *mcm2*_AA_

**Figure 3:**
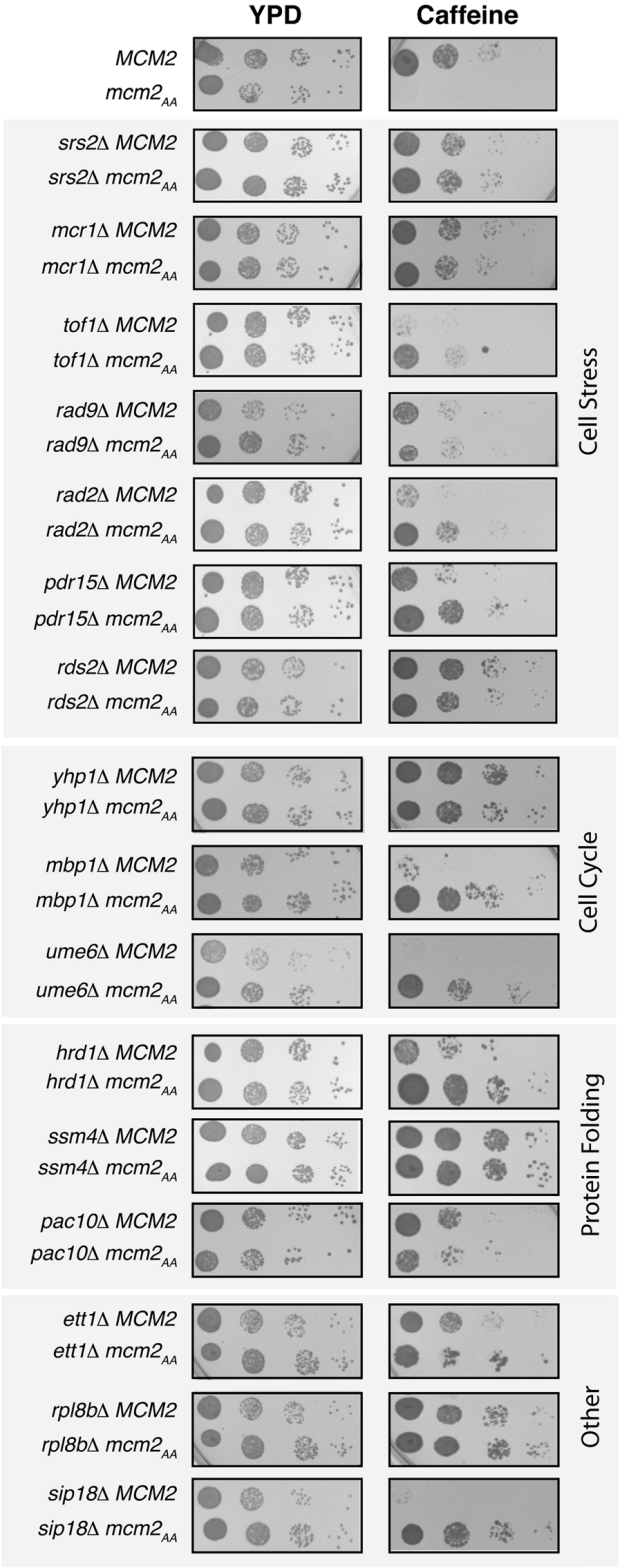
**Identification of gene deletions that suppress *mcm2***_AA_***.*** The growth of strains with deletions that suppress the caffeine sensitivity of *mcm2*_AA_ was compared on YPD and YPD with 15 mM caffeine. Approximately 5000 cells/ml and 10-fold serial dilutions were spotted and grown at 30°C.

As *mcm2*_AA_ is predicted to interfere in the response to replicative stress, second site mutations that decrease DNA damage or increase the capacity for DNA repair would be expected to act as suppressors. We therefore screened the haploid deletion strain collection for gene deletions that suppress the caffeine sensitivity of *mcm2*_AA_. Sensitivity to caffeine was chosen because of the strong phenotype it elicits with *mcm2*_AA_. Candidates were re-mated, isolated by tetrad dissection and re-tested on YPD with caffeine. Sixteen gene deletions were identified (Figure 3). We classified these genes by biological functions based on gene ontology annotations in the Saccharomyces Genome Database [37] as well as their reported functions in the literature (Table 2). These classifications yielded four groups of genes: cell stress, cell cycle, protein folding and “other” functions. Interestingly, half of the deletions, when independent of *mcm2*_AA_, were sensitive to caffeine indicating roles for these genes in response to caffeine. Four of these, *tof1Δmbp1Δume6Δ* and *sip18Δ* were as sensitive to caffeine as *mcm2*_AA_*.* Others, such as *rad9Δ, rad2Δ, pdr15Δ* and *hrd1Δ,* displayed an intermediate sensitivity. In addition, three showed decreased sensitivity to caffeine compared to wild type *MCM2* (*yhp1Δ, ssm4Δ* and *rpl8bΔ*). We also note that some of the deletions, such as *tof1Δ, rad9Δ* and *pac10Δ,* resulted in only partial suppression (Figure 3).

**Table 2:** **Suppressors of the caffeine sensitivity of *mcm2***_***AA***_

Gene	ORF	Function	GO
*srs2Δ*	YJL092C	DNA repair, helicase	1
*mcr1Δ*	YKL150W	Oxidative stress response	1
*tof1Δ*	YNL273W	Subunit of fork pausing complex	1,2,5
*rad9Δ*	YDR217C	Transmission of checkpoint signal	1,2,4,56
*rad2Δ*	YGR258C	Nucleotide excision repair	1,4,6
*pdr15Δ*	YDR406W	Cellular detoxification	1,3
*rds2Δ*	YPL133C	Transcription factor	1,6,7
*yhp1Δ*	YDR451C	Transcription factor/cell cycle	2,4,6
*mbp1Δ*	YDL056W	Transcription factor/cell cycle	2,4,6
*ume6Δ*	YDR207C	Transcription factor	2,4,5,6
*hrd1Δ*	YOL013C	Ubiquitin ligase/ER assoc. decay	8
*ssm4Δ*	YIL030C	Ubiquitin ligase/ER assoc. decay	8
*pac10Δ*	YGR078C	Protein folding	8
*sip18Δ*	YMR175W	Osmotic stress	9
*rpl8bΔ*	YLL045C	Ribosomal protein	9
*ett1Δ*	YOR051C	Translation termination	9

Genes are grouped by their GO terms as annotated in the *Saccharomyces cerevisiae* database [37]. Horizontal lines separate different classes. Gene ontology: (1) Response to cell stress/chemical stimuli (2) cell cycle, (3) transport, (4) RNA metabolic process, (5) signalling process/protein modification process, (6) transcription, (7) carbohydrate metabolic process, (8) ER-mediated degradation & protein-folding and (9) other.

**Figure 4:**
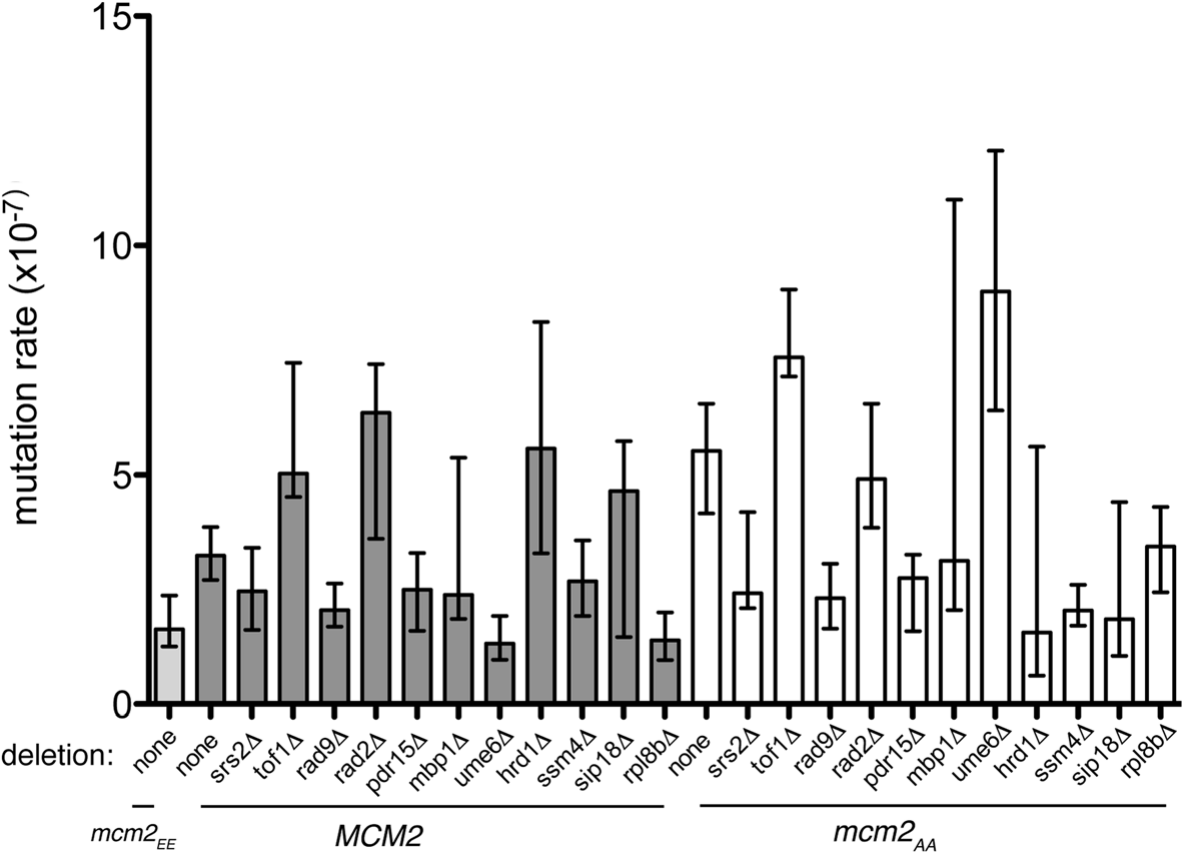
**Mutation rates of *mcm2 *alleles and strains with gene deletions that suppress *mcm2***_AA_**.** The mutation rates of *MCM2* and *mcm2*_AA_ strains with and without gene deletions that suppress *mcm2*_AA_ were calculated as described [39,40] using the *CAN1* forward mutation assay. Dark grey bars are the deletions in the *MCM2* background; white bars are with *mcm2*_AA_ and the light grey bar (far left) is the *mcm2*_EE_ strain. The error bars represent the upper and lower confidence limits (95%) of the mutation rates and were calculated from the 95% confidence limits of the median determined from the binomial distribution [40].

Our hypothesis predicts that deletions that suppress the caffeine sensitivity of *mcm2*_AA_ will also decrease the mutation rate in the *mcm2*_AA_ strain. Therefore, we repeated the *CAN1* forward mutation assay on a subset of the deletion strains. As shown in Figure 4, seven of the 11 deletions tested decreased the mutation rate, both with the deletion alone and in the presence of *mcm2*_AA_*.* The exceptions were *tof1Δrad2Δ* and *ume6Δ.* Deletion of *tof1* causes genomic instability [41-43] and *rad2Δ* is deficient in nucleotide excision repair [44]. Ume6 is involved in the expression of several genes and deletion of *ume6* increases homologous recombination [45-48].

**Figure 5:**
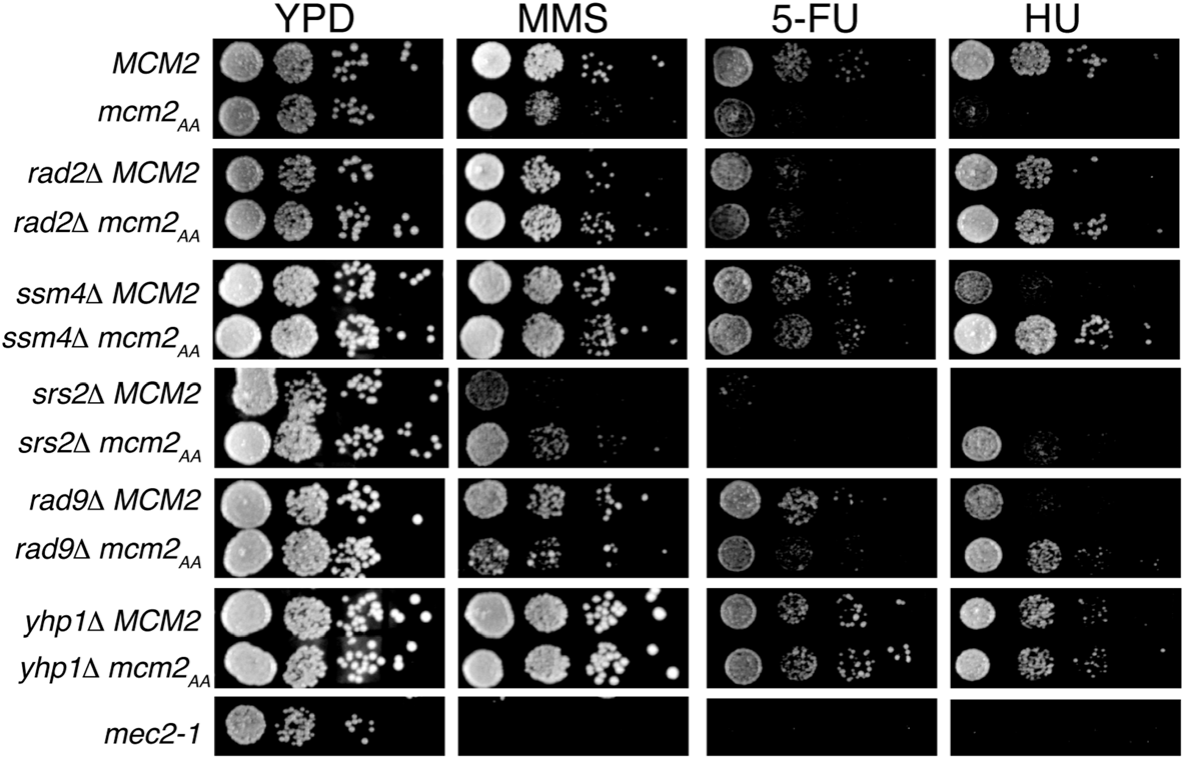
**Sensitivity of strains containing gene deletions that suppress *mcm2***_***AA ***_**to MMS, 5-FU and HU.** The growth of strains containing deletions that suppress the caffeine sensitivity of *mcm2*_AA_ was examined on YPD with and without 0.03% MMS, 400 μM 5-FU or 200 mM HU. Ten-fold serial dilutions of the strains were spotted on each of the plates and grown for the indicated times. Shown here are selected strains. The results with all of the suppressors are shown in Additional file 1.

To further evaluate the mechanisms by which the gene deletions suppress *mcm2*_AA_, we spotted strains containing the suppressor deletions and either *MCM2* or *mcm2*_AA_ onto YPD plates containing MMS, 5-FU and HU (Figure 5, Table 3 and Additional file 1). All of the deletions that suppressed the caffeine sensitivity of *mcm2*_AA_ also suppressed at least one other drug sensitivity of *mcm2*_AA_, exemplified by *rad2Δ* and *ssm4Δ* (Figure 5). Many of the deletions in the cell stress group also lead to sensitivity to these drugs in the *MCM2* background, thus likely accounting for the complex phenotypic patterns. For example, the *srs2* and *rad9* deletions do not suppress the sensitivity of *mcm2*_AA_ to MMS or 5-FU and only partially suppress on HU (Figure 5). Some of the deletions decrease sensitivity to the drugs in an otherwise wild type background*.* For example, the *yhp1Δ* strain grows faster than the wild type strain on plates containing MMS or 5-FU (Figure 5). This increased growth is also noted in the *mcm2*_AA_*yhp1Δ* strain. Therefore, the *yhp1* deletion likely functions non-specifically to suppress *mcm2*_AA_. Of note, Yhp1 is a transcriptional repressor that along with Yox1 is involved in the cyclic transcription of a set of genes that includes *MCM2-7*[49]. However, deletion of *yhp1* alone did not affect expression of *MCM3-lacZ* and had little or no effect on cell growth [49]. The *ett1Δ* deletion on MMS or HU and *sip18Δ* on HU similarly increase growth of cells containing the wild type and mutated *mcm2* alleles (Table 3 and Additional file 1).

**Table 3:** **Phenotypes of deletions that suppress the caffeine sensitivity of *mcm2***_***AA***_

	**Sensitivities**				**Suppression of *mcm2***_***AA***_				
mutation	caff	MMS	5-FU	HU	MMS	5-FU	HU	RFA foci	mutation rate
*srs2Δ*	R	S	S	S	-	-	+	+	+
*mcr1Δ*	R	R	S	S	-	+	+	ND	ND
*tof1Δ*	S	S	R	S	+	+	+/−	-	-
*rad9Δ*	S	S	R	S	-	-	+	+	+
*rad2Δ*	S	S	S	S	+	+	+	+	-
*pdr15Δ*	S	R	S	R	+	+	+	+	+
*rds2Δ*	R	S	S	S	+	+/−	+	ND	ND
*yhp1Δ*	R+	R+	R+	R	+	+	+	ND	ND
*mbp1Δ*	S	R	R	S	+	+	+	+	-
*ume6Δ*	S	R	R	R	+	+	-	+	-
*hrd1Δ*	S	S	R	S	+	+	+	+	+
*ssm4Δ*	R+	R	R	S	+	+	+	+	+
*pac10Δ*	R	S	S	S	+	+	+	ND	ND
*sip18Δ*	S	S	R	R+	+	+	+	+	+
*rpl8bΔ*	R+	S	S	S	+	+	+	+	+
*ett1Δ*	R	R+	R	R+	+	+	+	ND	ND

The sensitivities of the deletion strains (with wild type *MCM2*) to each drug is indicated by “R” for growth similar to wild type, “S” for growth slower than wild type or “R+” for better than wild type growth. For suppression of the phenotypes associated with *mcm2*_AA_, “-“indicates no suppression, “+” indicates suppression, “E” indicates epistasis and “ND” indicates that the test was not performed on that strain.

**Figure 6:**
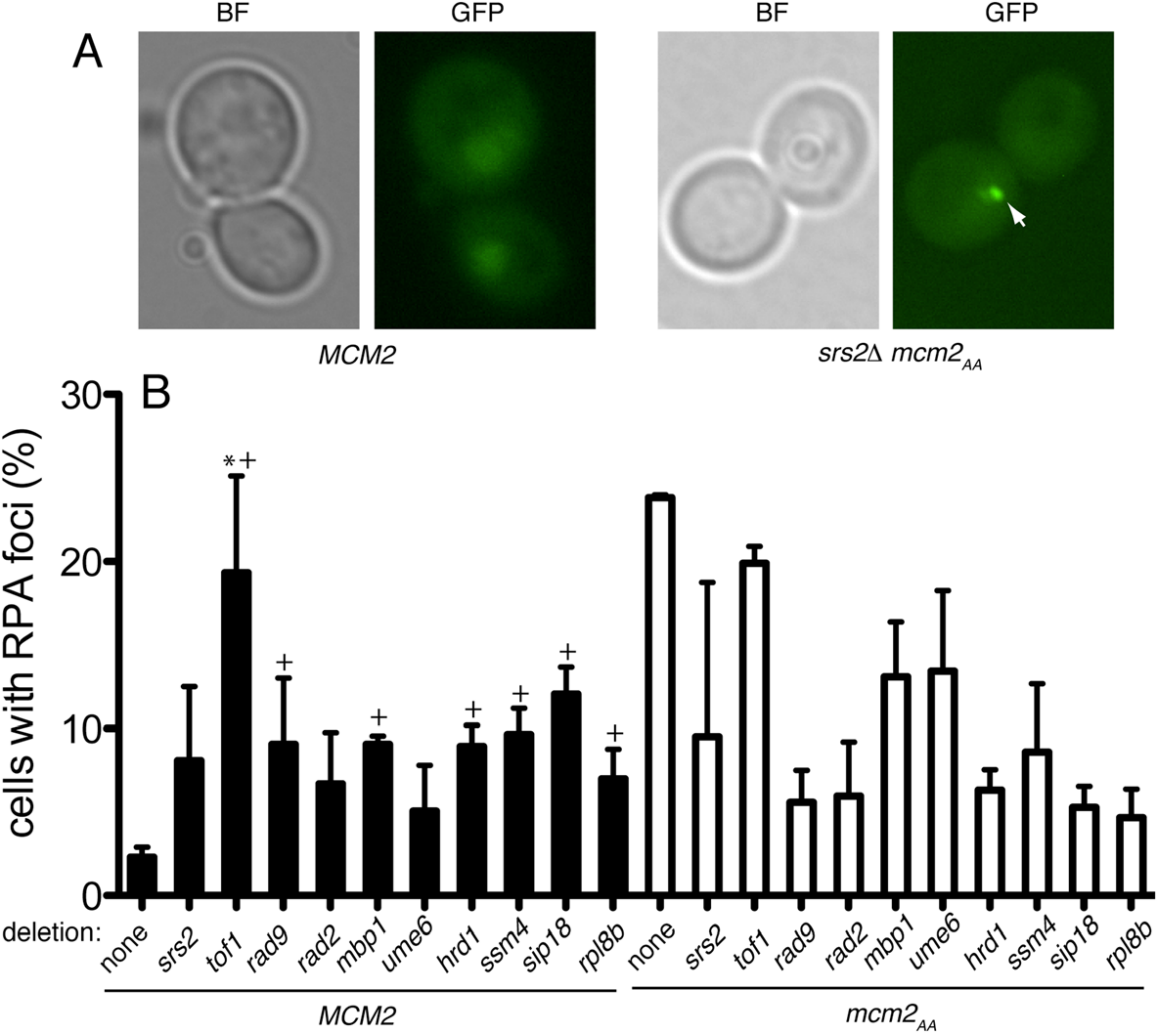
**RPA foci in the suppressor strains.** Cells with RPA foci were identified using GFP fused to Rpa1 as described [5]. **A)** An example of RPA foci is shown. Bright field (BF) and fluorescent (GFP) images are shown for *MCM2* (left panels) and *srs2*Δ *mcm2*_AA_ (right panels). A RPA focus in the *srs2*Δ *mcm2*_AA_ image is indicated by the white arrow. **B)** The percentage of cells containing RFA foci was measured in triplicate experiments and standard deviation was determined. The asterisks indicate strains that do not have statistically significant different frequency of RPA foci compared to *mcm2*_AA_ (p ≥ 0.05, Student’s unpaired *t*-test) while the crosses indicate strains that are statistically different from wild type (p < 0.05).

Previously, we observed that the *mcm2*_AA_ strain has a higher frequency of cells with RPA foci [5]. RPA is the single-stranded DNA binding protein and thus foci represent generation of single stranded DNA. In wild type cells, RPA is diffuse in the nucleus (Figure 6A). In a low percentage of wild type cells, RPA foci will appear. Since a higher frequency of cells contain foci when treated with DNA damaging agents, the foci are thought to represent ongoing repair processes or disruption of the replication fork, both of which generate stretches of single stranded DNA [30-32]. Interestingly, in cells containing *mcm2*_AA_, RPA foci appear in a much higher frequency of cells; ~20 percent (Figure 6A and B; [5]). As a means of determining the mechanisms by which the gene deletions suppress *mcm2*_AA_, we tested whether they also suppress the increased frequency of cells with RPA foci. We transformed a plasmid encoding GFP-tagged Rpa1 into a subset of the suppressor strains and scored each for cells with RPA foci (Figure 6B). In isolation, the gene deletions had a higher ratio of cells with RPA foci than wild type (Figure 6B) with the *tof1, rad9, mbp1, hrd1ssm4sip18* and *rpl8b* deletions having p values less than 0.05, reflecting the effect of these deletions on genomic stability. Despite this increase, deletion of most of the genes tested suppressed the increased frequency of RPA foci in the *mcm2*_AA_ strain; *srs2Δ* did not (p ≥ 0.05). Deletion of *tof1* also did not suppress *mcm2*_AA_. Indeed, *tof1Δ* in the wild type background greatly increases the frequency of cells with RPA foci. This increased frequency might be explained by the observation that disruption of the *tof1* ortholog in *S. pombe* (*swi1*) decouples polymerases, generating excess single stranded DNA [50-52].

## Discussion

Our findings suggest a role for phosphorylation of Mcm2 by DDK in response to replicative stress. Specifically, we demonstrate that the *mcm2*_AA_ strain is sensitive to drugs that cause replicative stress, has an increased mutation rate and that *mcm2*_AA_ interacts with genes involved in the response to replicative stress. Along with our previous study showing that phosphorylation of Mcm2 at S164 and S170 slows DNA unwinding and results in enhanced DNA binding by Mcm2-7 *in vitro*[5], our results lead to a model in which phosphorylation of Mcm2 slows DNA unwinding by Mcm2-7 and/or stabilizes the replication fork as part of the proper response to replicative stress.

When a replication fork encounters DNA damage such as a base lesion or a break in the DNA strand, synthesis by the replicative polymerases at that fork halts. A series of events must then occur for replication to proceed [53]. While double stranded DNA breaks, base damage or nucleotide depletion each induce the S phase checkpoint, which inhibits further initiation of DNA replication and stabilizes replication forks, the form of the response differs depending on the type of perturbation [54,55]. Given the sensitivity of the *mcm2*_AA_ strain to MMS and 5-FU, we propose that phosphorylation of Mcm2 by DDK is required to stabilize replication forks in response to DNA base damage. The lack of sensitivity to phleomycin with this strain suggests that Mcm2 phosphorylation may not be required in response to double strand breaks. That DDK phosphorylation would trigger Mcm2 participation in the response to replicative stress is not surprising given DDK participates in responses to DNA damage and replicative stress and is a target of Rad53 during the S phase checkpoint [56-60].

### Genetic interactions with mcm2_AA_

**Figure 7:**
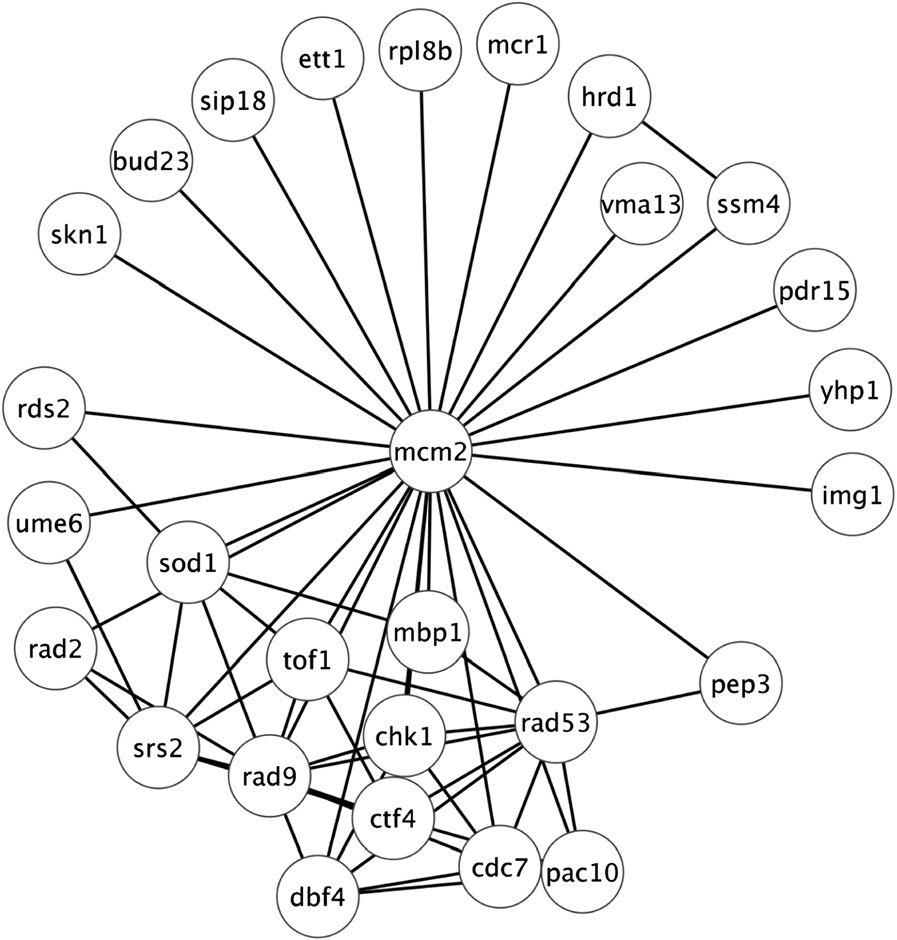
**Interactions amongst *mcm2***_***AA ***_**interactors.** A map of interactions amongst the genes connected with *mcm2*_AA_ was generated using Cytoscape 2.8.2 [61,62]. Physical and genetic interactions annotated on SGD [37] were used to generate the map. Also included are interactions within this group with *CDC7* and *DBF4*, the genes encoding the components of DDK.

The genetic interactions with *mcm2*_AA_ are most consistent with a requirement for Mcm2 phosphorylation in response to disruption of the replication fork. The effect of the suppressing deletions can be explained as either decreasing spontaneous DNA damage, which would otherwise disrupt replication forks or increasing the capacity for rescue of disrupted forks by recombination. In contrast, the deletions that result in synthetic lethal interactions increase spontaneous DNA damage, perturb the replication fork and/or are required for checkpoint responses. Mapping the interactions within the 25 genes connected to *mcm2*_AA_ indicates that about 15 form a network independently of *mcm2* (Figure 7)*.* Most of the interacting genes have roles in response to DNA damage and replicative stress. Interestingly, several of the genes interact with *CDC7* and *DBF4,* the genes encoding the two components of DDK (Figure 7).

Of the 25 genetic interactions with *mcm2* that we isolated, only one was previously identified. Deletion of *rad9* has a synthetic lethal interaction with *mcm2-1*, a temperature sensitive allele [63]. Interestingly, here *rad9Δ* suppresses some, but not all, of the phenotypes associated with *mcm2*_AA_*.* The difference may result from allele specificity; *mcm2-1* is expected to affect Mcm2’s essential role in Mcm2-7 function whereas the *mcm2*_AA_ allele affects Mcm2 activity in response to replicative stress.

Deletion of three genes, *HRD1, SSM4* and *PAC10* whose products are involved in targeting misfolded proteins for degradation [64,65], suppress *mcm2*_AA_*.* One possible mechanism for these suppressors is that misfolded proteins induce the unfolded protein response (UPR) which in turn provides protection against reactive oxygen species which can damage DNA [66]. However, the mechanism of suppression is more complex since *mcm2*_AA_ is still sensitive to caffeine or MMS in the presence of tunicamycin, which induces the UPR (data not shown).

### Regulating helicase progression in response to replicative stress

We propose that phosphorylation of Mcm2 by DDK is required in response to replicative stress. This role is not recognition of damage or replication fork collapse since the S phase checkpoint is intact at least up to Rad53 phosphorylation. Furthermore, *mcm2*_AA_ has synthetic lethal interactions with *chk1Δ* and *mec2-1*, mutations in the genes encoding the checkpoint effector kinases in yeast [2,67-69]. Synthetic lethal interactions often indicate function of the interacting genes in parallel pathways. Based on the previously observed biochemical activities of Mcm2-7 with DDK-phosphorylated or phosphomimetic Mcm2 [5], we surmise that phosphorylation of Mcm2 may stabilize Mcm2-7 on DNA and/or slow the helicase. DNA helicases are predicted to contact DNA through the sugar phosphate backbone, not the bases [70]. Therefore, unlike replicative polymerases that stall at sites with missing or damaged bases, the helicase will continue unwinding DNA and may decouple from the polymerase. Indeed single stranded DNA generated by decoupling of helicase from the replicative polymerases is proposed to generate a checkpoint response [71-77]. Decoupling appears to be regulated so that the helicase does not advance too far from the rest of the replisome, leading to complete disassembly of replication forks [78]. One role for Mcm2 phosphorylation may be to slow the helicase so that it does not proceed too far ahead of the replicative polymerases. In this model, the accumulation of RPA foci in the *mcm2*_AA_ strain is not due to spontaneous decoupling of the helicase from polymerase but rather is due to DNA damage triggering decoupling of the helicase.

The idea that replicative stress triggers a requirement for Mcm2 phosphorylation by DDK is supported by the synthetic lethal interaction of *mcm2*_AA_ with deletions that lead to genomic instability. Examples include *ctf4Δ* and *img1Δ.* Ctf4 is involved in sister chromatid cohesion and is integral to the RPC [16,36,79-81]. Deletion of *img1* leads to loss of functional mitochondria which in turn leads to genomic instability [34,35]. Increased DNA damage is also noted in strains with increased levels of ROS, such as *sod1Δ*[33] and *vma13Δ*[82,83], both of which are synthetically lethal with *mcm2*_AA_. Rad9 is important for transmission of checkpoint signalling with deletion of *rad9* resulting in cells that fail to arrest in response to DNA damage [84,85]. The increased homologous recombination noted with *srs2Δrad2Δ* and *ume6Δ* may suppress *mcm2*_AA_ by providing a means to resolve stalled replication forks [45,86-88]. Finally, the model that Mcm2 phosphorylation may be required to slow replication forks is supported by the ability of *tof1Δ* to suppress defects of *mcm2*_AA_ only in the presence of genotoxic agents (*tof1Δ* does not suppress the increased mutation rate or increased frequency of RPA foci). Lack of Tof1, a member of the RPC, slows replication forks [43], however this occurs only in the presence of replicative stress, such as seen in the presence of genotoxic agents [42].

## Conclusions

Phosphorylation of Mcm2 by DDK is required for the proper response to replicative stress, but not to induce a checkpoint. This phosphorylation event likely slows the Mcm2-7 helicase and/or stabilizes replication forks. In the absence of Mcm2 phosphorylation, the mutation rate is increased.

## Methods

### Materials

Caffeine, HU and MMS were purchased from Sigma Aldrich (99% purity); geneticin (G418) from either United States Biological or Santa Cruz Biotechnology; phleomycin from Santa Cruz Biotechnology; 5-FU from Nutritional Biochemicals Corp. YPD is 1% yeast extract, 2% peptone and 2% *D*-glucose. The yeast strains MDY167 (*MCM2-URA3*), MDY169 (*mcm2*_AA_*-URA3*) and MDY191 (*mcm2*_EE_*-URA3*) are described in Stead et al. [5] and the *mec2-1* strain in [24].

### Synthetic lethal screen

The genetic screens were a modification of SGA analysis [89]. Manipulation of the gene arrays was performed manually using a 3.18-mm 48-pinner tool (V&P Scientific*,* San Diego, CA). Screens were performed using MDY169 (*MAT***α***mcm2*_AA_*URA3*) or MDY167 (*MAT***α***MCM2-URA3*[5]) mated with the haploid yeast Magic Marker deletion collection generated from the diploid strain collection (Open Biosystems; [90]). Diploids were selected and then sporulated before selecting haploid cells that contained a gene deletion (Kan^R^) and *mcm2*_AA_ or *MCM2* (Ura^+^). Plates were scanned on a HP Scanjet 3970 and colony size was compared using ImageQuant TL (GE Healthcare). A synthetic lethal or slow growth (SSL) phenotype was assigned if the colony size was smaller in the *mcm2*_AA_ cross than in the *MCM2* cross. A false positive rate (growth when the gene deletion is reported as lethal in SGD [37]) was roughly 5% and is similar to false positive rates reported previously [89]. From this initial screen, we compiled a list of 234 genes that had SSL interactions with *mcm2*_AA_. Next, the candidates were retested from the mating step in triplicate. Forty-one SSL interactions were identified in this step. The deletions were then re-tested for genetic interactions with *mcm2*_AA_ by re-mating followed by tetrad dissection. SSL interactions were verified if Ura + and G418^R^ spore progeny were never recovered or grew slowly relative to the corresponding single mutations.

### Screen for suppressors of caffeine sensitivity

A screen to isolate deletions that suppress the caffeine sensitivity of *mcm2*_AA_ was performed by pinning the haploid *mcm2*_AA_ strains that also contained a gene deletion (generated as described above) to YPD containing 15 mM caffeine. The plates were incubated at 30°C for 4 days, imaged and then quantified. A spot was determined to contain a deletion that suppresses *mcm2*_AA_ when its size was larger than the spot size of the *mcm2*_AA_ strain. The deletion of 369 genes suppressed the growth defect of *mcm2*_AA_ in caffeine in the initial screen. These candidate deletions were re-mated in triplicate, haploids generated and retested for sensitivity to caffeine, resulting in 86 strains containing a deletion that potentially suppresses *mcm2*_AA_. The 86 were strains re-mated, sporulated and tetrads dissected. A G418 resistant, Ura + colony was identified, grown to saturation and 10-fold serial dilutions were spotted onto YPD with and without 15 mM caffeine.

### Mutation rate assay

The forward mutation assay was performed as described in [91]. Briefly, at least 20 colonies were inoculated into 10 x 1 ml YPD and the cultures grown overnight at 30°C to 1–2 x 10^8^ cells/ml. Each culture was diluted to approximately 200 cells/1 ml YPD and grown to 1–2 x 10^8^ cells/ml. Cells were then plated (~ 10^7^) on CM-Arg plates containing 25 μg canavanine/ml and appropriate dilutions were made before plating on YPD. Colonies on each plate were counted to determine the number of canavanine resistant cells per 10^7^ cells. The mutation rate was calculated using the method of the median [39,40].

## Authors' contributions

BES carried out the screens and drug assays with assistance from CJB and MKS and wrote a draft of the manuscript. MJD did the microscopy, mutation assays, Rad53 blot and edited the manuscript with CJB. All authors read and approved the final manuscript.

## Supplementary Material

Additional file 1 Stead et al., Supplemental Data. Click here for file
