# Supplementary material for: Mcm2 phosphorylation and the response to replicative stress
Source: BMC Genet. 2012 May 7;13:36. doi: 10.1186/1471-2156-13-36 (PMC3517340; doi:10.1186/1471-2156-13-36)
Supplement: Additional file 1 — Stead et al., Supplemental Data. [file 1471-2156-13-36-S1.pdf]

Stead et al., Supplemental Data

Supplemental Figure 1: Tetrad Dissections . A. Representative spore colonies from tetrad dissections. Each column represents a single tetrad. Synthetic lethal interactions of *mcm2*$_{AA}$ with the indication mutations are demonstrated by the lack of spore colonies containing both mutations. B. Similarly, smaller colony sizes were observed with spore colonies from tetrad dissection of strains containing *mcm2AA* and the indicated deletion. In addition, slower growth was observed with the deletion in a *mcm2*$_{AA}$ background when ten fold serial dilutions of a saturated culture of the indicated colonies were spotted on YPD plates.

Supplemental Figure 2: Sensitivity of the suppressors of caffeine sensitivity to other genotoxic agents. Strains with a gene deletion that suppresses the sensitivity of *mcm2*$_{AA}$ to caffeine were tested for sensitivity to 0.03 % MMS, 400 μM 5-FU or 200 mM HU by spotting 10-fold serial dilutions of the strains on media containing the drugs and on YPD. Growth was compared to the *MCM2* and *mcm2*$_{AA}$ strains. A strain containing the *mec2-1* allele was also examined to control for the quality of the plates. Results of this figure are summarized in Table 3.

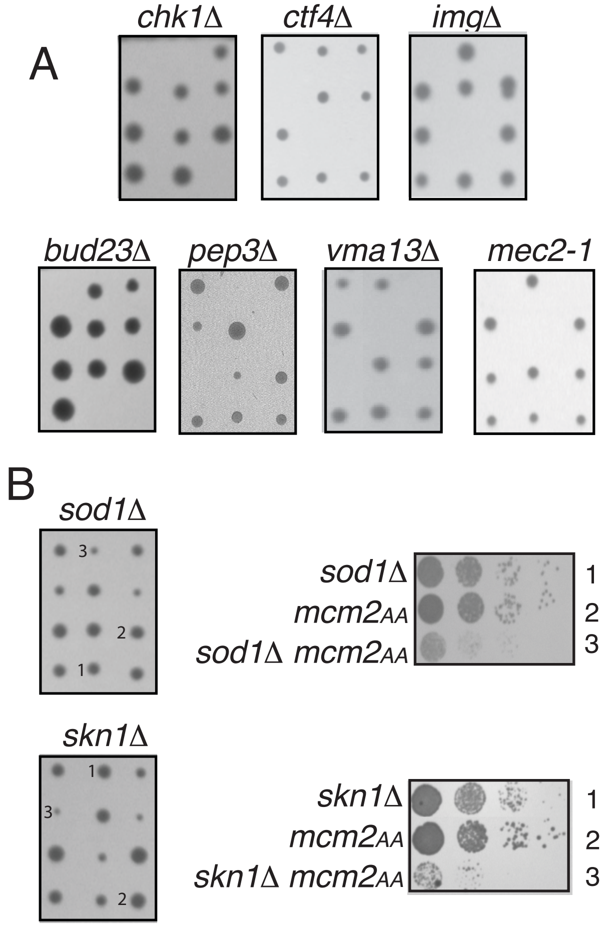

Stead et al., Supplemental Figure 1

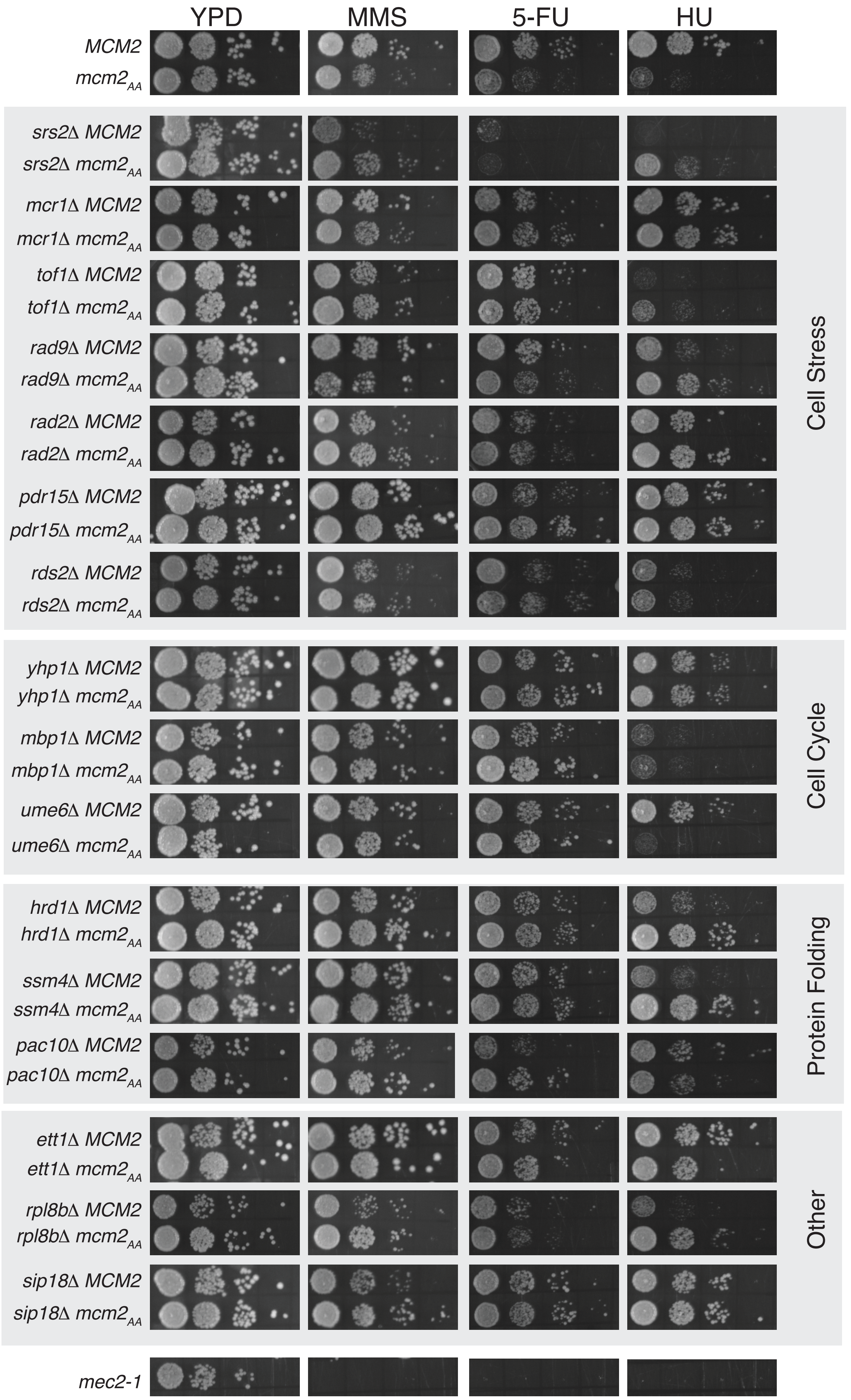

Stead et al., Supplemental Figure 2
